# Visible Light-Driven GO/TiO_2_-CA Nano-Photocatalytic Membranes: Assessment of Photocatalytic Response, Antifouling Character and Self-Cleaning Ability

**DOI:** 10.3390/nano11082021

**Published:** 2021-08-08

**Authors:** Rooha Khurram, Aroosa Javed, Ruihua Ke, Cheng Lena, Zhan Wang

**Affiliations:** 1 Beijing Key Laboratory for Green Catalysis and Separation, Department of Chemistry and Chemical Engineering, Beijing University of Technology, Beijing 100124, China; rooha_khurram@hotmail.com (R.K.); lenacheng_bjut@outlook.com (C.L.); 2 Department of Chemistry, School of Natural Sciences (S.N.S.), NUST, H-12, Islamabad 44000, Pakistan; aroosa.899@gmail.com; 3 School of Ecological Construction and Environmental Protection, Jiangxi Environmental Engineering Vocational College, Ganzhou 341002, China

**Keywords:** environmental science, Graphene oxide (GO), Titania (TiO_2_), Photocatalytic membranes, photo-degradation of MO, self-cleaning and anti-fouling ability, clean technology and engineering

## Abstract

Photocatalysis and membrane technology in a single unit is an ideal strategy for the development of wastewater treatment systems. In this work, novel GO (x wt%)/TiO_2_-CA hybrid membranes have been synthesized via a facile non-solvent induced phase inversion technique. The strategy aimed to address the following dilemmas: (1) Effective utilization of visible light and minimize e^−^/h^+^ recombination; (2) Enhanced separation capability and superior anti-fouling and self-cleaning ability. The experimental results reveal that the integration of nano-composite (GO/TiO_2_) boosts the membrane properties when compared to pristine CA and single photocatalyst employed membrane (GO-CA and TiO_2_-CA). The effect of GO content on the properties of the photocatalytic membrane has been determined by utilizing three different ratios of GO, viz. 0.5 wt%, 1 wt%, and 2 wt% designated as NC(1)-CA, NC(2)-CA, and NC(3)-CA, respectively. Amongst them, NC(3)-CA membrane showed state-of-the-art performance with an elevated photocatalytic response (four times higher than pristine CA membrane) toward methyl orange. Moreover, the water flux of NC(3)-CA membrane is 613 L/m^2^h, approximately three times higher than bare CA membrane (297 L/m^2^h), while keeping the MO rejection high (96.6%). Besides, fouling experiments presented the lowest total and fouling resistance ratios and a higher flux recovery ratio (91.78%) for the NC(3)-CA membrane, which endows the membrane with higher anti-fouling and self-cleaning properties. Thus, NC(3)-CA membrane outperforms the other as synthesized membranes in terms of separation efficiency, visible light photo-degradation of pollutant, anti-fouling and self-cleaning ability. Therefore, NC(3)-CA membrane is considered as the next generation membrane for exhibiting great potential for the wastewater treatment applications.

## 1. Introduction

Global water needs are estimated to reach $6.7 trillion by 2030 and $22.6 trillion by 2050 [1]. Nearly half the world’s population already lives with water scarcity. Owing to the growing demand for water, the future potential for treating wastewater is enormous [2]. Consequently, to attain the Millennium Development Goals, priority actions must be undertaken to reduce untreated wastewater discharges. Hitherto, several gray infrastructure systems, such as membrane technology [3], adsorption [4], biomembrane process [5], ion exchange [6], activated sludge process [7,8,9], coagulation and sedimentation [10,11], electrochemical technology [12], have come to play a significant role in wastewater treatment challenges. Specifically, membrane technology has achieved state-of-the-art performance in water treatment technology. However, today such gray infrastructure system are struggling to meet our demands, as well as at risk of miserable failure in a changing world, owing to inherent shortcomings, including membrane fouling, lower permeation flux and membrane life, high energy consumption, and treatment cost [13].

Similarly, photocatalytic technology represents sustainable, efficient, and green infrastructure for wastewater treatment. Sunlight is considered an inexhaustible, eco-friendly energy source and has utilized the deprivation of dye pollutants. However, to separate nanosized photocatalyst, the photocatalytic system required an additional separation step. Otherwise, there is a considerable chance of secondary pollution. To address these compelling challenges strategically blending green infrastructure with grey technology bring forth to meet the research goals. 

A photocatalytic membrane (PM) integrates the “gray infrastructure” with “green infrastructure” and conquers the loopholes of both techniques. Photocatalytic membranes incorporate photocatalysis (green infrastructure) and membrane separation (gray infrastructure) in a single unit [14]. Under sunlight irradiation, a photocatalyst generates OH, $O_{2}^{-}$ and H_2_O_2_, known as reactive oxygen species, which deteriorate the contaminant in the feed solution, thereby warding off cake layer formation on the membrane surface, diminish pore-blocking, as well as hampering membrane fouling [15,16]. In the meantime, the membrane substrate acts as a selective barrier for nano-photocatalysts, thus reducing the time needed for photocatalyst recovery and reusability [17,18,19]. PMs overcome photocatalysis and membrane technology defects by their innovative designs and most worthwhile multimodal functionalities. 

With the best literature survey, Zhang et al. [20] and Choi et al. [21] proposed the concept of photocatalytic membranes in 2006. In the report, the authors have functionally modified the ceramic membrane surface with the TiO_2_ based photocatalytic layer for water treatment. Due to this coupling process, the membrane surface prevents degraded organic contaminants from permeating through the membrane. Meanwhile, they mitigate membrane fouling due to the coherent decomposition of pollutants through photocatalysis. TiO_2_ can be affirmed as the archetypical photocatalyst, a suitable synonym for photocatalysis. Owing to inherent characteristics, including vigorous catalytic activity, high stability in the chemical or photochemical environment, a wide range of pH, relatively cheap cost, low toxicity, enhanced hydrophilicity, and suitable self-cleaning property, one can tailor the permeation performance of the membrane. It has been extensively utilized in the domain of photocatalytic membranes [14]. However, the UV active photocatalyst uses only 5% of the solar spectrum, rapid electron-hole recombination, and demonstrates a greater tendency for aggregation, which impedes its feasibility for practical application in photocatalytic membranes [22,23]. Several approaches have been considered so far, like the formation of heterojunctions with other semiconductors [24,25], doping (metal or non-metal), [26] heterostructure formation with carbon-based materials, including quantum dots (QDs), graphene oxide (GO) [27], and (rGO) [28] to conquer the significant concerns. Among them, GO has attracted considerable interest from the material researchers. The desirable properties in the membrane filtration and photocatalysis domain mainly include superior hydrophilicity, good dispersion in the polymer matrix, high surface area, and excellent electron acceptor and donor capability, thereby showing excellent photocatalytic responses [29,30,31]. Coupling GO and TiO_2_ is considered the practical way to tailor the shortcomings associated with TiO_2_ [32,33,34,35]. Extensive research towards supplementing the GO/TiO_2_ nano-composites on membrane surfaces has been reported to endow membranes with desired characters [36,37,38,39,40,41]. Recently, authors reported the modification of polyethersulfone (PES) membrane surface with GO/TiO_2_ nano-composites with enhanced water purification [42,43]. The PVDF membrane surface [44] and CA membrane surface [36] have also been modified with graphene/titania nano-composite employment, suggesting great potential for water treatment. Nonetheless, membrane surface modification methodology has some drawbacks, mainly the loss of particles from the membrane when subjected to long filtration operation [45]. 

Although persistent efforts have been made over the past few years, the development of photocatalytic membranes towards the proficient treatment of water pollutants is still in its infancy. This work employed the most common, simple, yet efficient, versatile, and intriguing non-solvent induced phase separation (NIPS)-blending approach to synthesize membranes. This technique will effectively anchor the as prepared nano-composites into the polymer matrix. Firstly, GO/TiO_2_ nano-photocatalysts were synthesized by a one-pot hydrothermal approach. Secondly, by utilizing NIPS appraoch GO/TiO_2_ photocatalysts integrated cellulose acetate membranes (GO/TiO_2_-CA hybrid membranes) were synthesized. Thirdly, nano-photocatalysts and hybrid membranes were separately characterized via various techniques. Lastly, the performance of hybrid membranes was evaluated by permeation and photodecomposition of methyl orange (MO). To the best of our knowledge, the research concerning GO/TiO_2_-CA hybrid membrane, the effect of GO content in GO/TiO_2_ nano-composite on membrane performance, elucidation of underlying in-depth study of photocatalytic mechanism, assessment of the self-cleaning and anti-fouling performance of these hybrid membranes has never been reported before.

## 2. Experimental Section

### 2.1. Chemicals

For the preparation of bare TiO_2_ nano-photocatalyst, titanium (IV) tetraisopropoxide (TTIP), 2-propanol, and nitric acid (HNO_3_) were purchased from Merck. The raw materials utilized for the graphene oxide synthesis include graphite powder, sodium nitrate (NaNO_3_), potassium permanganate (KMnO_4_) with purity >99%, sulphuric acid with purity >98%, 30% aq. Hydrogen peroxide (H_2_O_2_) and 6M hydrochloric acid (HCl) were purchased from Beijing chemical engineering factory. In this study, the cellulose acetate (CA) used for pristine membrane synthesis was purchased from ande membrane separation technology & engineering (Beijing) Co., Ltd. Herein, NMP was used as a solvent and PVP to prevent the aggregation of TiO_2_ nanoparticles was bought from Tianjin Fuchen Chemical Reagent Factory. The modal pollutant methyl orange (MO) was purchased from Shanghai Maikun Chemical Factory. All the chemicals and reagents were of analytical grade and used without further purification.

### 2.2. Synthesis

(a) TiO_2_ synthesis

**Figure 1:**
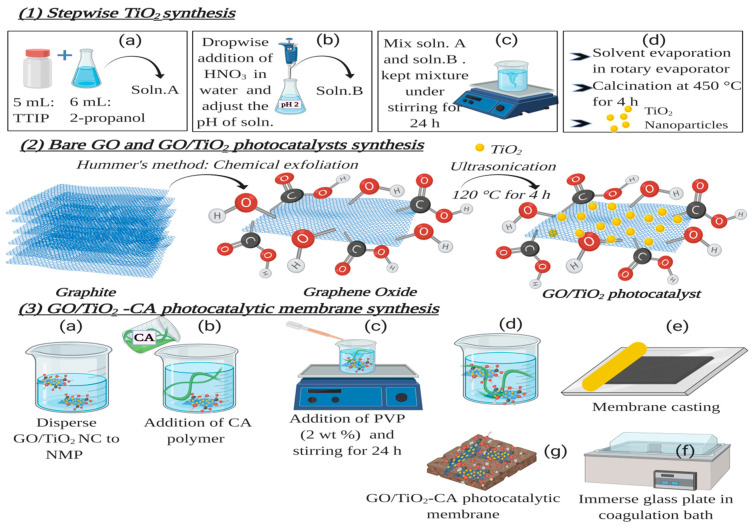
Synthesis schemes (**1**) TiO_2_ synthesis (**2**) Bare GO and GO/TiO_2_ synthesis (**3**) GO/TiO_2_-CA photocatalytic membrane synthesis.

Titanium (IV) tetraisopropoxide (TTIP) was the primary starting material. The homogenous mixture of TTIP (5 mL) and 2-propanol (6 mL) was prepared under stirring for 30 min. This mixture is termed as solution A. Afterwards, **a** few drops of nitric acid were added to water (85 mL) to adjust its pH to 2, and it was designated as solution B. Finally, the drop wise addition of Solution A into solution B was carried out under vigorous stirring. At RT, the mixture was stirred continuously for 24 h. After that, the dried powder was obtained by evaporating the solvent in a rotary evaporator. Then, this powder was calcined at 450 °C for 4 h in the furnace and then ground for 1 h. The resultant product was stored for further analysis. The synthesis scheme is presented in Figure 1.

(b) GO synthesis

Graphene oxide was synthesized utilizing Hummers’ method (Figure 1). In this method, graphite powder (1 g), conc.H_2_SO_4_ (23 mL), and sodium nitrate (0.5 g) were mixed in a 500 mL flask. This flask was kept in an ice bath under continuous stirring. When the temperature approached 0 °C, potassium permanganate (3 g) was added very slowly to the suspension with constant stirring. As the addition of potassium permanganate is an exothermic reaction, so the addition of potassium permanganate to the suspension was carefully controlled. So that the temperature doesn’t go above 15 °C. Then, this mixture was kept under stirring for 1 h at 30 °C. After stirring, deionized water (150 mL) was added to it very slowly, and the color of the mixture was changed to brown. Once again, the mixture was kept under stirring for 2 h. Subsequently, after a 2 h delay, 30% H_2_O_2_ aq. solution was added. This addition results in a change of color from brown to yellow. After that, the mixture was kept under continuous stirring for 15 min followed by sonication for an hour. Finally, through the centrifugation of mixture at 4000 rpm for 40 min, the GO was obtained. Then, GO was washed with 10% HCl after DI washing. The product obtained was dried under vacuum at 60 °C.

(c) GO/TiO_2_ nano-composite synthesis

For the synthesis of GO/TiO_2_ nano-composite facile, a one-pot hydrothermal approach was utilized (Figure 1). In a typical procedure, GO was dispersed in water (80 mL) via ultrasonication. Afterward, a certain amount of TiO_2_ NPs was added to the dispersion. To attain homogenous suspension, the dispersion, as mentioned earlier, was kept under continuous stirring for 2 h. Subsequently, the mixture was held in a heating oven for 4 h at 120 °C. The final product of GO/TiO_2_ nano-composites was collected via washing 3 times with distilled water through centrifugation. The sample is dried by vacuum drying for 24 h. To explore the effect of different GO content, all nano-photocatalysts and membranes were synthesized with varying amounts of GO (0.5, 1.0, and 2.0 wt%) and categorized as NC-x (x = 1, 2, 3 respectively).

(d) GO/TiO_2_ nano-photocatalytic membranes synthesis

All the nano-photocatalytic and pristine membranes follow the same phase inversion synthesis procedure as presented in Figure 1. The detailed UF hybrid membrane synthesis procedure includes: (1) 1 h ultrasonication of the varied concentration of NCs (0.1, 1.0, and 2 wt%) in a specified amount of NMP, (2) To this uniform dispersion, add CA, (3) After the uniform dissolution of CA polymer, a certain amount (2 wt%) of Polyvinylpyrrolidone (PVP) as porogen was added and to acquire homogeneous solution followed by stirring for 24 hrs. (4) To eradicate the bubbles, the polymer mixed solutions were kept at room temperature for one day. Once the degassing phenomenon is complete, the membrane was cast on the clean glass plate, and then immersed in a coagulation bath immediately filled with water at RT. The consequent membranes were then washed to remove the residual solvents and kept in DI water for further use. The composition matrixes of diverse membranes are presented in Table 1.

### 2.3. Characterization of Photocatalysts

The scanning electron microscope is one of the essential characterization techniques used to get information about the topography, morphology, and elemental composition. SEM images were taken by JEOL JSM-6460. The elemental composition of all the samples was determined using an analytical technique known as EDS. SEM is equipped with EDS. EDS collects the data from SEM. X-ray diffraction (XRD, Bruker D8Discover) with CuKα radiation (1.54059Å) is a non-destructive analytical technique used to analyze crystalline materials. XRD data can be used to determine the crystallite size by using the Scherrer Equation (1).

(1) D=Kλ/βcosθ

Brunnauer–Emmett–Teller analysis (BET, NOVA 2200 e Quanta Chrome, Chicago, The United states of America (USA)) was utilized to determine the surface area of all nano-photocatalysts. It employs liquid nitrogen for adsorption, followed by degassing at 300 for several hours. Helium gas is utilized for creating an inert atmosphere. Fourier transform infrared spectroscopy (FTIR) is a spectroscopic technique used to identify organic and inorganic compounds. Diffuse reflectance spectroscopy (DRS) is an optical method commonly used to describe the electronic behavior present in the structure of materials. UV/VIS diffuse spectrophotometer (C-640UV–Vis, Japan model) was used to measure the reflectance spectra of the samples, including TiO_2_, GO, and NC-x nano-composites. The reflectance data were manipulated into a tauc plot employing the Kubelka–Munk Equation (2).

(2) $$\alpha h\nu^{1/2}=K\left(h\nu -Eg\right)$$

### 2.4. Characterization of Hybrid Membranes

Scanning electron microscopy (SEM, FEI Quanta, Amsterdam, Holland) was employed to determine the cross-sectional and top surface morphologies of all nano-photocatalytic membranes (CA, GO-CA, TiO_2_-CA, NC(1)-CA, NC(2)-CA, and NC(3)-CA). The samples were prepared in liquid Nitrogen followed by gold-sputtering. The FTIR analysis was carried out using Nicolet 6700, Thermo Electron Corp., Chicago, The United states of America (USA) was utilized for the identification of the organic/inorganic compounds in all the samples. Moreover, to understand the hydrophilicity of all prepared membranes, water contact angle (G10, Kruss, Hamburg, Germany) was measured utilizing a sessile drop method. Each contact angle value is the average of three replicates to minimize the measurement error. The membrane porosity ($P_{O}$) was calculated using the following equation:

(3) $$P_{O}=\frac{\left(W_{1}-W_{2}\right)/\rho_{W}}{\left(W_{1}-W_{2}\right)/\rho_{W}+W_{2}/\rho_{P}}$$

where, $P_{O}$ represent membrane porosity,$W_{1}$, $W_{2}$ shows the dry and wet weight of membrane (g), $\rho_{P}$ and $\rho_{W}$ indicates the polymer and water (g/cm^3^) density. 

### 2.5. Configuration, Rejection, and Permeation of Hybrid Membranes

**Figure 2:**
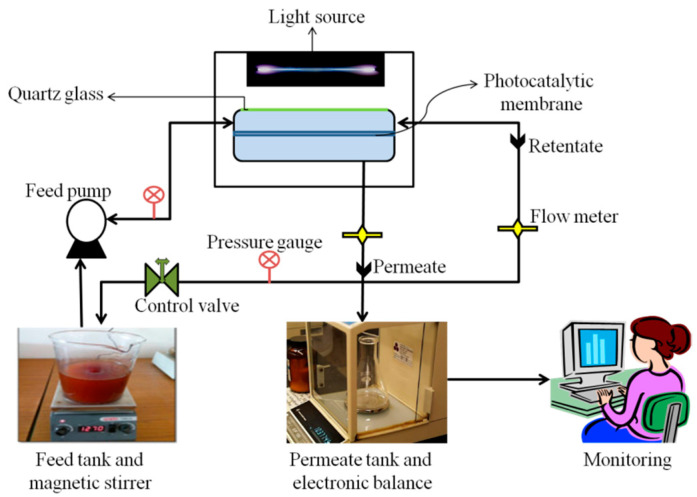
Self-designed continuous cross-flow setup utilized in this study.

To examine the flux performance and anti-fouling analysis of the photocatalytic membranes whole experimental study was carried out in a self-designed continuous cross-flow configuration (Figure 2). The filtration setup is well equipped with membrane cells, having an effective membrane filtration area of 19.6 cm^2^, feed pumps, flow meter, pressure gauge, pressure control valve, and monitoring system. The filtration cell is made up of quartz glass to permit the transmission of the light source. Firstly, pure water flux (PWF, $J_{w}$) was investigated by maintaining feed pressure at 0.2 MPa for almost 1 h. Secondly, to calculate the flux of MO solution, 50 mg/L of MO solution was utilized as feed solution. *J*_MO_ was calculated using the method like pure water flux but under the 0.1 MPa pressure at room temperature. During the experimental study, pressure and flow rate were kept constant. The formula for the calculation of $J_{w}$ and *J*_MO_ was Equation (4):

(4) $$J_{w}==\frac{V}{At}$$

where, V, A, and t represents permeate volume (L), the surface area of the membrane (m^2^), as well as the time needed to acquire the volume through the membrane (h). The rejections (R) of MO solution were investigated by ascertaining the MO concentration in the feed and permeate solution using (UV-9200) UV-spectrophotometer at a maximum wavelength of 464 nm with the employment of Equation (5).

(5) $$R=\frac{C_{f}-C_{p}}{C_{f}}\times 100\%$$

where, $C_{p}$ represents the concentration of permeate solution (mg/L) while $C_{f}$ represents the concentrations of feed solution (mg/L), respectively.

### 2.6. Performance Evaluation of Hybrid Membranes

#### 2.6.1. Photocatalytic Property of Membranes 

Firstly, the piece of the membrane was set up on a glass slide. Afterward, the glass slide was imbued/immersed in a petri dish containing 50 mL of MO solution. The lamp was mounted just above the petri dish. The light source was a Xe lamp (visible light irradiation, 100 mW cm^−2^, wavelength >400 nm, UV filter). Before turning on a lamp, the solution was kept in darkness for 1 h to establish adsorption equilibrium. The photocatalytic activity of pure CA membrane, GO-CA membranes, TiO_2_-CA membranes, and GO/TiO_2_-PVDF membranes with different GO content labeled as NC(x)-CA were assessed using UV-spectrophotometer (UV-9200) by observing the decrement in the concentration of MO at regular interval. 

#### 2.6.2. Anti-Fouling Performance and Self-Cleaning Ability of Various Membranes

A four-step filtration procedure was carried out for the evaluation of the anti-fouling performance of the hybrid membranes.

(1) Calculation of stable PWF (*J_w_*).
(2) Calculation of the MO solution flux (*J_MO_*): 1 wt% of MO (50 mg/mL) was utilized as feed solution to calculate MO flux for 120 min.
(3) To measure the flux of the rinsed membranes (*J_RM_*): After finding the flux of MO solution, the membranes were washed with deionized water for 1 min to remove the foulant. At this time, the flux of rinsed membranes was calculated and designated as *J_RM_*.
(4) Finally, we measure the flux of cleaned membranes (*J_CM_*): To further remove the pollutants, the above membranes were kept under the light source for 30 min. Subsequently, the flux of cleaned membranes was calculated and designated as *J_CM_*.

The last step was carried out without and with a light source under identical conditions for comparative analysis. Nonetheless, steps 1, 2, and 3 were conducted without light. To evaluate the filtration resistance, Equation (6) is used to find out the fouling resistance of the used model:

(6) $$J_{MO}=\frac{\Delta P}{\mu R_{t}}$$

where Δ*P*, *μ* (1.005 × 10^−3^ Pa s) and *R_t_* refers to trans-membrane pressure (TMP, 0.1 MPa), the viscosity of water, and the sum of the resistances, respectively.

(7) $$R_{t}=R_{m}+R_{d}+R_{a}+R_{f}$$

*R_m_* (intrinsic membrane resistance) was calculated by the formula:

(8) $$R_{m}=\frac{\Delta P}{\mu J_{w}}\;$$

The resistance that happened to owe to the cake layer formation on the surface of the membrane is called deposition resistance (*R_d_*). *R_d_* is calculated as:

(9) $$R_{d}=R_{t}-\frac{\Delta P}{\mu J_{RM}}\;$$

Resistance occurs due to the strong adsorption of dye molecule on membrane surface or pores are known as adsorption resistance (*R_a_*). It is measured as:

(10) $$R_{a}=R_{t}-R_{d}-\frac{\Delta P}{\mu J_{CM}}\;$$

This resistance is calculated by exposing the fouled membrane (after washing) under the light source for 40 min.

Resistance due to the pore blocking or irreversible adsorption of MO foulant called fouling resistance (*R_f_*) and is calculated as follows:

(11) $$R_{f}=R_{t}-R_{d}-R_{a}-R_{m}$$

#### 2.6.3. Self-Cleaning Performance of Photocatalytic Membranes

The self-cleaning ability of different hybrid membranes was calculated by analyzing more parameters about the fouling process, including Flux recovery rate *(FRR*), total fouling ratio (*R_t_*), irreversible (*R_ir_*), and reversible (*R_r_*) were calculated using Equations (12)–(15). The hybrid membranes that qualify for outreach maximum recovery in the performance of membrane following fouling confirm its higher self-cleaning ability.

(12) $$FRR=\frac{J_{CM}}{J_{w}}\times 100\%$$

(13) $$R_{t}=\left(1-\frac{J_{MO}}{J_{w}}\right)\times 100\%$$

(14) $$R_{ir}=\left(\frac{J_{CM\;}-J_{MO}}{J_{w}}\right)\times 100\%$$

(15) $$R_{r}=\left(\frac{J_{w\;}-J_{CM}}{J_{w}}\right)\times 100\%$$

where, *J_w_* is the stable PWF, *J_CM_* is the flux of cleaned membranes after light irradiation, and *J_MO_* is the flux of MO solution. 

## 3. Results and Discussion

### 3.1. GO/TiO_2_ Nano-Photocatalysts Characterization 

**Figure 3:**
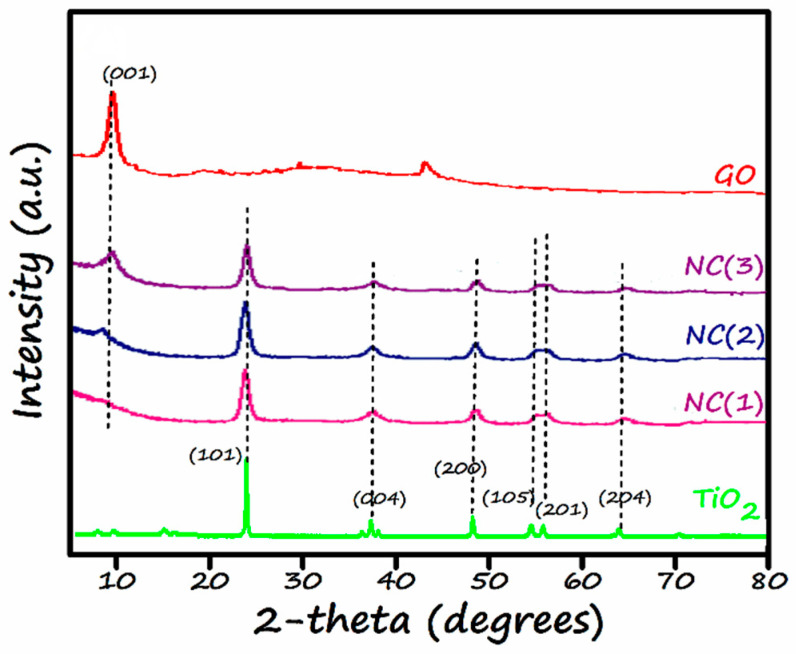
XRD patterns of TiO_2_, GO, NC(1), NC(2) and, NC(3).

JEOL-JDX-II, an X-ray diffractometer, was utilized for the XRD analysis. Figure 3 represents the XRD spectra of TiO_2_, GO, and GO/TiO_2_ nano-composites (NCx) with varying content of GO (x = 0.5, 1, 2) designated as NC(1), NC(2), and NC(3). The XRD pattern of TiO_2_ showed diffraction peaks at 25.3°, 37.8°, 48.9°, 55.1°, 58.3°, and 62.7° having their lattice planes (101), (004), (200), (105), (201), and (204), which were well matched with the standard JCPDS card number 21-1272. The bare TiO_2_ has a tetragonal structure with only an anatase phase. The presence of no rutile phase is attributed to the low-temperature synthesis procedure [46]. 

XRD pattern of graphene oxide shows a diffraction peak at 2 θ = 10° which correspond to the diffraction plane (002), which shows that graphite has been successfully oxidized to GO. The appearance of a small peak at 2 θ = 41° may be due to the presence of some amount of unreacted graphite. Moreover, the absence of a peak around 26° confirms the successful oxidation process has occurred towards GO formation. Owing to the presence of oxygen-containing functionalities, the interlayer spacing of GO (0.84 nm) was observed to be higher than graphite (0.34 nm) [47].

The XRD patterns of nano-composites (NCx) where x = 1, 2, 3 have been shown in Figure 3. These XRD patterns attest that nano-composites showed a peak at 2 θ = 10°, which means that graphene oxide successfully maintained its identity. However, the intensity of the corresponding peak has been increased gradually. This refers to a relative mass percentage increase of GO from NC (1) to NC (3). The lowest intensity (almost no peak) of GO in NC-1 is due to minimum content of GO (only 0.5%) in the respective nanocomposite. Moreover, all of these nano-composites show characteristic peaks at 25.3°, 37.4°, 48.07°, 55.4°, 58.3° and, 62.7°. The presence of these peaks confirms the existence of TiO_2_ in its anatase form. The bare photocatalysts, viz. GO and TiO_2_, showed highly intense and sharp peaks, thus confirming their crystalline nature. Whereas the nano-composites spectra clearly show the slight broadness and very slight shift towards the lower angle in the diffraction peaks, they undoubtedly reveal the presence of chemical interaction between TiO_2_ and GO.

Crystallite sizes of all the catalysts were determined using Scherer’s formula [24], as shown in Table 2. The crystallite size of all the prepared photocatalysts lies in the range of 10–20 nm. 

**Figure 4:**
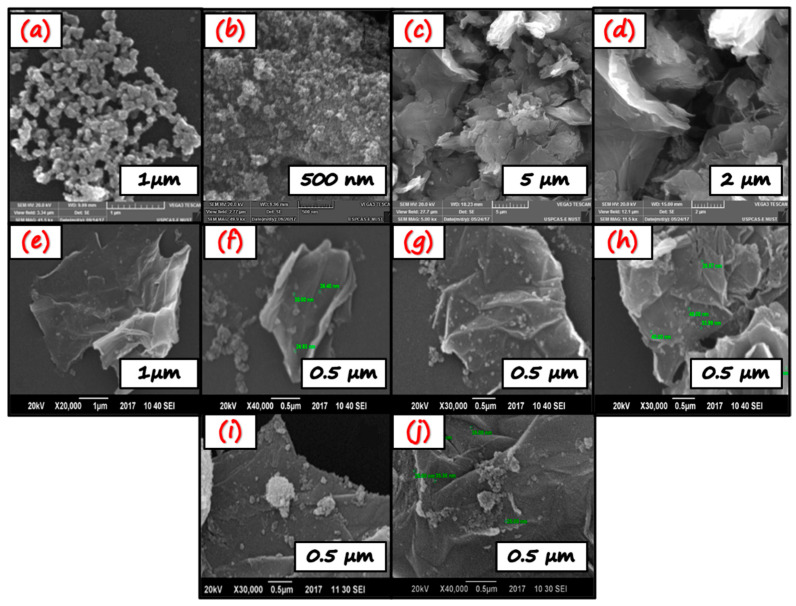
SEM analysis of TiO_2_ (**a**,**b**), GO (**c**,**d**), NC-1 (**e**,**f**), NC-2 (**g**,**h**), NC-3 (**i**,**j**).

SEM observed the morphology of samples with different scales. Figure 4a,b depict the morphology of TiO_2_, which is found to be spherical agglomerated nanoparticles. Figure 4c,d represent the SEM images of graphene oxide. The SEM images indicate that graphene oxide contains aggregated crumpled multilayer sheets because of the introduction of oxidizing functional groups. The GO sheets have irregular and folded structures formed as a result of stacking. This morphology is beneficial to the growth of TiO_2_. As shown in Figure 4e–j, numerous TiO_2_ NPs are uniformly and evenly distributed/decorated over the GO sheets. As the GO content increases, from NC-1 to NC-3, a slight change in morphology was observed. As the GO layers are curled in NC-1 while in NC-3, they constitute less stacking and have few layers. The change in morphology from curled to loose and less stacking may be attributed to the introduction of GO, which hampers the aggregation of TiO_2_ nanoparticles, especially in NC-3. Thereby, the surface area is increased. Herein, the spherical morphology of TiO_2_ nanoparticles integrating with loose GO sheets designs the surface properties and surface area and tunes the electronic structure, i.e., improves the photocatalytic activity due to the more active visible light spectrum [48]. The TiO_2_ exhibit dispersed morphology in the case of all nano-composites. The well-distributed TiO_2_ on the graphene oxide (GO) planes is evident in the successful loading of TiO_2_ on GO planes. This interaction between TiO_2_ nanoparticles and GO can promote charge transfer between them. Thus, they play a vital role in the enhanced photocatalytic response. All the SEM images show that the nanoparticles have an average particle size below 40 nm, spherical, and evenly distributed.

The BET surface area of TiO_2_, GO/TiO_2_ (0.5 wt%), GO/TiO_2_ (1 wt%), and GO/TiO_2_ (2 wt%) are shown in Table 2. The results show that bare TiO_2_ has the lowest surface area and GO/TiO_2_ (2 wt%) nano-composites exhibit the highest surface area. The high content of GO in the composite is responsible for the increase in the surface area. SEM images are consistent with these findings. 

**Figure 5:**
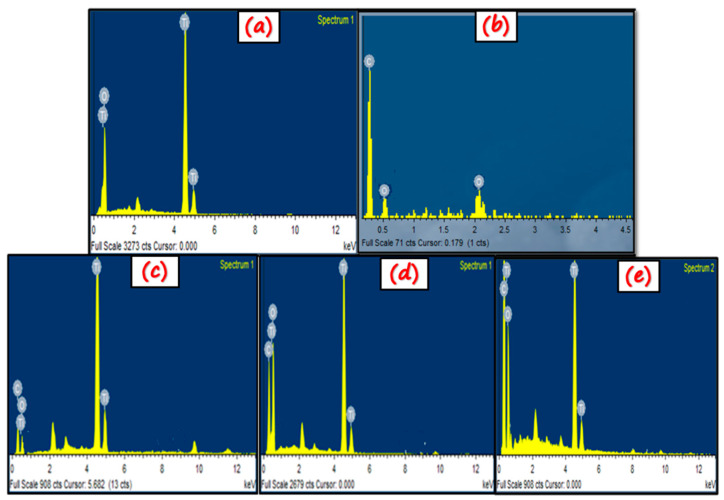
EDX spectra of (**a**) TiO_2_, (**b**) GO, (**c**) NC-1, (**d**) NC-2, and (**e**) NC-3.

EDX is utilized to find the elemental composition and purity of the sample. The presence of every element in the sample is confirmed by its specific peak in the respective graph (Figure 5). The EDX results of TiO_2_ (Figure 5a) clearly show the signals for titanium and oxygen. Figure 5b shows the signals of oxygen and carbon, which represents the GO sample. However, in nano-composites Figure 5c,d, the amount of titanium decreases while carbon and oxygen content increases from NC-1 to NC-3. This might be attributed to the increase in GO content from NC-1 to NC-3.

Moreover, in all the EDX spectra, there exists no extra peak except the concerned elements. This shows the impurity-free synthesis of all the nano-photocatalysts. The atomic % of each element that forms a particular photocatalyst, for instance, the atomic % of Ti and O in TiO_2_, atomic % of C and O in GO, and the atomic % of Ti, C, and O in GO/TiO_2_ nano-composites has been given in Table 2. 

**Figure 6:**
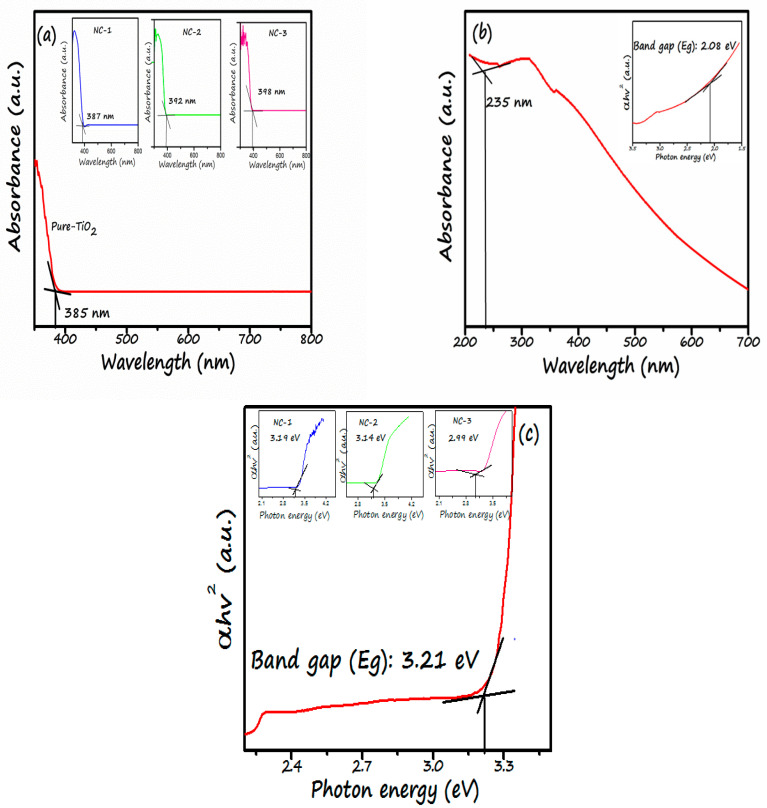
DRS spectra of (**a**) Absorption edge of Bare TiO_2_ (insets shows the zoom-in plot showing absorption edge of TiO_2_ in NC-1, NC-2 and, NC-3) (**b**) Absorption edge of GO (inset shows the Bandgap of GO), (**c**) Bandgap of bare TiO_2_ (insets shows the zoom-in plot showing band gap of TiO_2_ in NC-1, NC-2 and, NC-3).

The bandgap value is the most crucial factor in evaluating the photocatalytic activity of any photocatalyst. Reflectance measurements obtained from DRS are converted to absorption coefficient using The Kubelka–Munk function [25]. From Figure 6a, TiO_2_ shows an absorption edge at 385 nm, therefore absorb UV radiations from the solar spectrum. However, the UV part constitutes only 4% of the spectrum [48]. The photocatalyst should show absorption edge in the visible light region to utilize a wide range of the spectrum. In our study, the TiO_2_ forms heterostructure with GO as confirmed by XRD, IR, SEM, and EDX. This TiO_2_/GO nano-composite extends the absorption in the visible part of the spectrum. Thus, they exhibit an enhanced photocatalytic response. Graphene oxide (GO) shows evidence of high absorption in both visible and UV regions. The leading absorption edge of GO was observed at 235 nm. While the band gap observed was 2.08 eV as in Figure 6b. As for the various TiO_2_/GO nano-composites, as the GO content increases, visible light absorption increases. Exploiting the sunlight/effective absorption in the visible region implies higher efficiency towards photocatalytic purposes.

**Figure 7:**
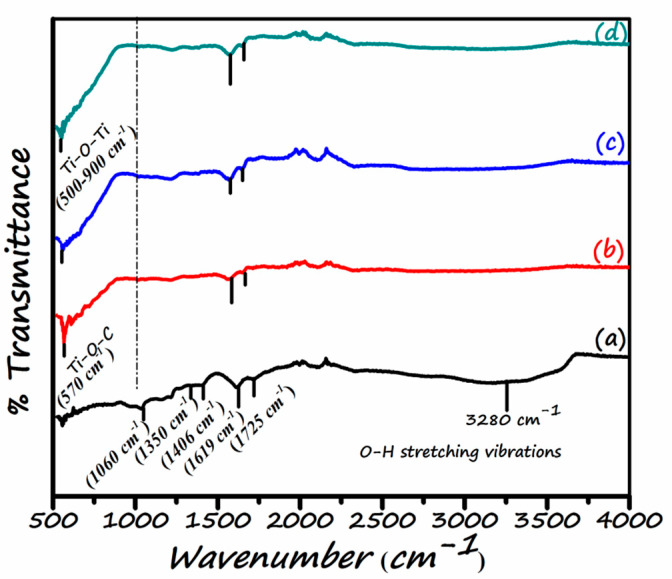
FT-IR spectra of (**a**) GO (**b**) NC-1 (**c**) NC-2 (**d**) NC-3.

The bandgap of all the samples was calculated using the tauc equation: αhv = A(hv − Eg)^2^ [24]. By plotting graph between (αhv)^2^ against hv. The band gap value of all the photocatalysts are depicted in Figure 6 and Table 1. Figure 6a insets show zoom-in plots for the nano-composites, which demonstrate that the absorption edge of titania has shifted towards the lower region. This means a redshift has been observed, viz. 387 nm, 392 nm, 398 nm, respectively, therefore extending the absorption of light in the visible region. The figure illustrates very little shift in the absorption edge. This might be credited to the increase of GO content in a low amount (only 0.5%). This shift in the extended absorption visible region is due to the formation of Ti-O-C chemical bonding, which is supplementarily verified from the FT-IR spectra (Figure 7b–d). The GO has a significant effect on the bandgap of TiO_2_. TiO_2_ band gap is decreased viz. 3.19, 3.14 and, 2.99 for NC-1, NC-2, and, NC-3 respectively, with the increase in GO content as depicted in Figure 6c. 

Table 2 summarizes all the prepared photocatalysts’ precise results, including percentage composition found by EDX, crystallite size calculated using the XRD values, particle size from SEM, and the bandgap (eV) value ascertained from DRS data, as well as the surface area estimated through BET analysis.

FT-IR spectroscopy is carried out for the understanding of bonding characteristics of functionalities forming photocatalyst. Figure 7 demonstrates the FT-IR spectra of all the samples. Various strong absorption bands of oxygenated functional groups are present in the spectrum of graphene oxide Figure 7a. The characteristic peaks appeared at 1060 cm^−1^, 1350 cm^−1^, 1406 cm^−1^, 1619 cm^−1^, 1725 cm^−1^, and 3407 cm^−1^ correspond to alkoxy/alkoxide C–O, the stretching vibration of epoxy/ether (C–O–C), carboxy O–H, aromatic skeletal vibration of C=C and H–O–H bend, the carboxy/carbonyl (C=O) stretching vibration and O–H stretching vibrations of the C–OH groups and H_2_O respectively [39,40]. The FT-IR spectra substantiate the formation of GO/TiO_2_ nano-composites. The broad absorption band between 448 and 1000 cm^−1^ is considered as the combined stretching vibrations of Ti-O bond originating from Ti–O–Ti network in the TiO_2_ spectra [49,50,51,52] and Ti-O-C bonds (around 570 cm^−1^) [53,54]. The Ti-O-C bond also represents the successful formation of GO/TiO_2_. This bond indicate that the TiO_2_ nanoparticles are successfully form heterojunction with GO sheets through the electrostatic interaction between the function groups of GO with the hydroxyl group of TiO_2_ [55,56]. Moreover, in FTIR spectra of three nanocomposites, the decrease in the intensities of typical absorption band or disappearance of bands can be seen clearly in contrast to GO. However, the absorption band at 1620 cm^−1^ represents the C=C stretching of the skeletal vibration of the graphene. This indicates that graphene is not entirely removed in the nano-composites. This fact is supported by the existence of a Ti-O-C bond in all the nano-composites. This depicts that the Ti-O-C bond is strong enough to not disturb the linkage between two components during hydrothermal synthesis. As the GO content increases, the width and intensity of absorption bands increase, implying the photocatalytic membrane’s enhanced hydrophilicity. The spectra of NC-3 (Figure 7d) demonstrate that the strength of oxygen containing functional groups is almost diminished/disappeared, suggesting the reduction of oxygen containing functional groups after the recombination of GO and TiO_2_ hydrothermally [57]. 

### 3.2. GO/TiO_2_-CA Photocatalytic Membrane Characterization

**Figure 8:**
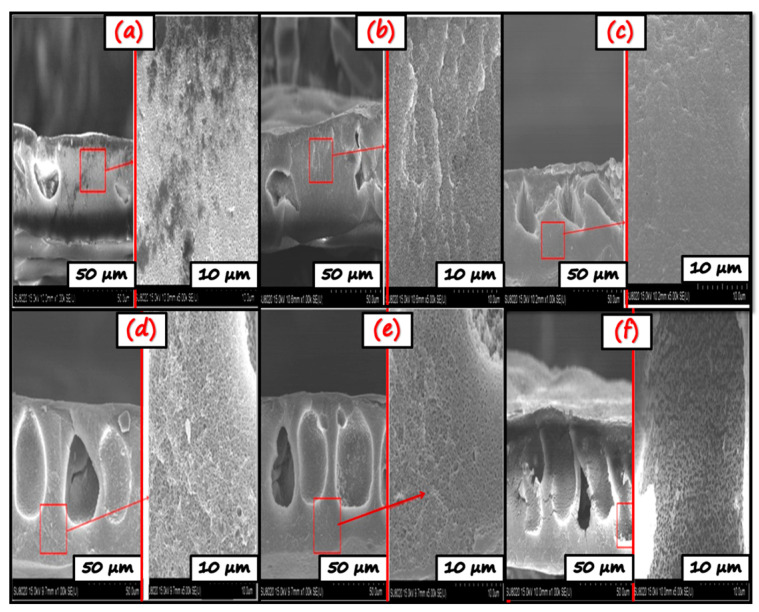
SEM cross-section morphology of (**a**) CA, (**b**) TiO_2_-CA, (**c**) GO-CA, (**d**) NC(1)-CA, (**e**) NC(2)-CA and, (**f**) NC(3)-CA.

Figure 8 shows cross-section morphology of all the prepared bare and hybrid photocatalytic membranes. SEM image of the bare CA membrane depict the porous and compact structure. This is because hydrophilic additives and PVP leach out into the non-solvent phase when the casted membrane is immersed into the water bath, resulting in the polymer’s coagulation into the non-woven sheet. Thereby, the interaction of membrane polymer and solvent resulted in the generation of a more porous asymmetric structure. Even after the prolonged stay of the membrane in non-solvent, some PVP may remain in the polymer matrix; this could be due to the polymer-polymer interaction. Afterward, GO/TiO_2_ nano-photocatalysts are employed in the membrane matrix. GO/TiO_2_ nano-photocatalysts, due to their hydrophilicity, tries to come out of the membrane. However, due to some entangled PVP (being amphiphilic), it holds the GO/TiO_2_ nano-photocatalysts in the membrane matrix. This leads to enhancing porosity and permeate flux as justified by permeation results. With the addition of NC-1, NC-2, and NC-3, surface roughness, a loose porous structure, and more finger-like projections increase, greatly influencing the water flux [58]. As shown in Figure 8, with the increase in GO content, the finger-like porous channel structure increases progressively. It might be ascribed to the faster exchange rate of solvent and non-solvent due to GO’s high hydrophilicity because of hydroxyl and carbonyl groups in GO. It is reported that with a higher exchange rate, additional finger-like porous channels are observed in the membrane [59]. Pure water flux and contact angle studies support this reason for an increase in hydrophilicity with NC-3 nano-photocatalyst in the membrane matrix due to the higher content of GO in it.

**Figure 9:**
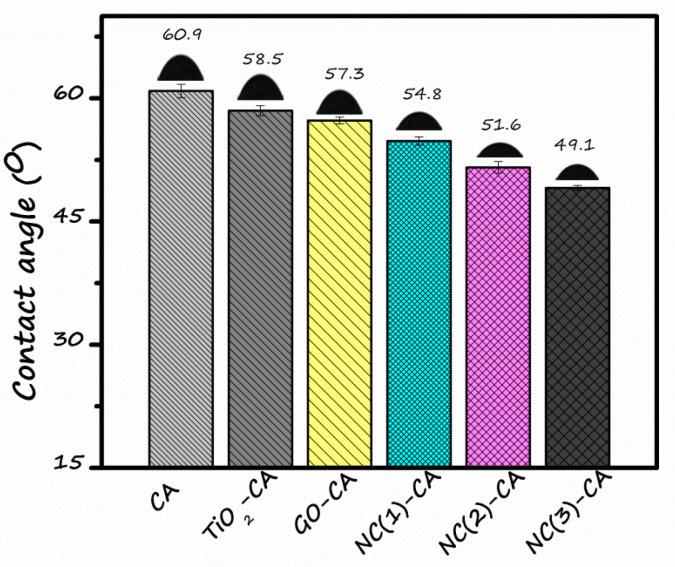
Water contact angle (WCA) measurement of various membranes.

Wettability measurements of the membrane were assessed by water contact angle (WCA). WCA is determined by sessile drop method. Figure 9 depicts the contact angle of the bare CA membrane (60.9°) was highest, demonstrating its hydrophobic nature. Although TiO_2_ nanoparticles are hydrophilic, the GO-CA still showed more hydrophilic character. It might be credited to the unique sheet structure of GO possessing a large number of hydroxyl groups, which impart a more hydrophilic character to the membrane [60]. When GO/TiO_2_ nano-photocatalysts with different GO content is employed, the contact angle value decreases progressively. The contact angle of NC(1)-CA, NC(2)-CA, and NC(3)-CA membrane is 54.8°, 51.6°, and 49.1°, respectively. This supports the observation that the introduction of nanocomposite increases the hydrophilic property of membranes owing to the higher content of hydrophilic species, i.e., GO/TiO_2_. Therefore, the NC-3 membrane has the lowest water contact angle (49.1°), and is accordingly highly hydrophilic. The increased hydrophilicity of hybrid membrane is subjected to two reasons. Firstly, during the phase inversion process, the decrement in the interface energy resulting in the spontaneous travel of nano-photocatalysts towards the interface of membrane/water [61]. Secondly, since contact angle is calculated using the sessile drop technique, sunlight is also employed for a few seconds, which triggers the production of reactive oxygen species, viz. OH and $O_{2}^{-}$ radical anions are generated by excitation of photocatalysts by absorbing light [39]. It further reduces the contact angle value to its smaller extent due to the photo-induced hydrophilicity of GO/TiO_2_. Moreover, the modification of membrane with GO/TiO_2_ nanocomposites increases the anti-fouling property due to the generation of ROSs details, as discussed in later sections [62]. 

**Figure 10:**
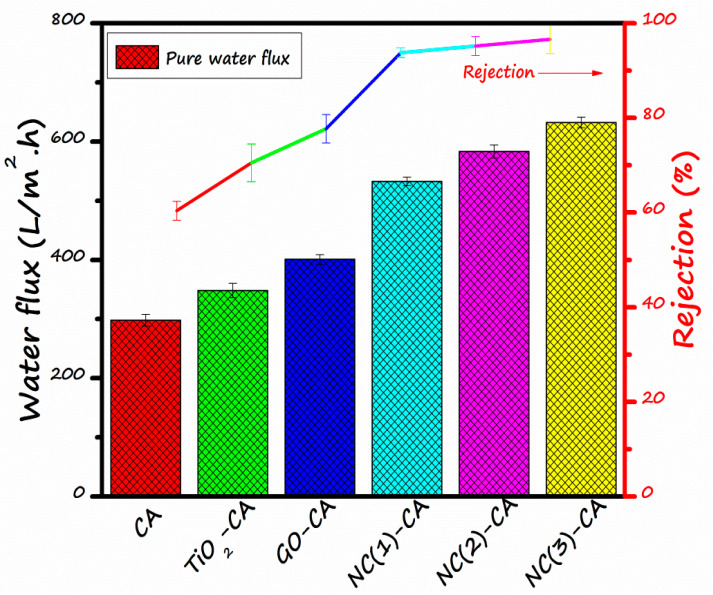
Pure water flux (**left** *y*-axis) and rejection (**right** *y*-axis) of several membranes.

Porosity (%) is one of the essential characterization tools in the membrane separation domain as it provides information about the void space in the polymer matrix. This study utilizes gravimetric analysis for the porosity measurement. The nanocomposite membrane showed higher porosity value than the bare CA membrane. The visual representation of the more porous structure of nanocomposite employed membranes is presented in SEM results, justifying the enhanced porosity (Figure 8). With the addition of 0.5% GO, the general porosity value has increased. The porosity value follows the sequence NC(3)-CA>NC(2)-CA>NC(1)-CA>CA, which corresponds to the values of 75.18% > 65.98% > 54.98% > 21.37%, respectively. Tentatively, the presence of PVP in the composition of the membrane matrix increases thermodynamic instability. This affects the structural properties of the membrane by altering the porosity [63]. However, GO/TiO_2_ NC’s addition to the casting solution works as a cherry to the top, further decreasing the thermodynamic stability, thus leading to the formation of more porous channels on the membrane surface. The increased porosity is another significant factor affecting the water flux, as presented in Figure 10. The supplement of membrane matrix with the nanocomposite exhibiting higher GO content increases the thickness of the membrane, which directly affects the separation performance of the membrane. In this study, the membrane with a higher amount of GO, i.e., NC(3)-GO, has high separation performance and pollutant removal tendency, as described in later sections.

Figure 10 illustrates the MO rejection and pure water flux (PWF) of various membranes. The results demonstrate the very typical trend that the pure water permeation flux and the rejection of MO dye are greater for GO/TiO_2_ nanocomposite employed hybrid membranes than bare CA, TiO_2_-CA or GO-CA membranes. In agreement with Figure 10, the water flux value of NC(3)-CA, NC(2)-CA, NC(1)-CA, GO-CA, TiO_2_-CA, and bare CA membrane was around 613(L/m^2^h), 595(L/m^2^h), 570(L/m^2^h), 400(L/m^2^h), 320(L/m^2^h), and 297(L/m^2^h), respectively. Results demonstrate that the permeation flux of bare CA is the lowest. While the pure water flux of the GO-CA membrane is superior to the TiO_2_-CA membrane, the GO membrane has more finger-like channels, higher porosity, and more excellent hydrophilicity than the TiO_2_ membrane. This fact is consistent with SEM results and contact angle measurement. However, the permeation flux of NC(3)-CA is the highest. This increase in the pure water flux is credited to: (1) the accelerated exchange between solvent and non-solvent during phase inversion, which led to more porous finger-like channels in the hybrid membranes responsible for the highest pure water flux [64]. This explanation is supported by SEM results. Moreover, the increased porosity of nano-photocatalytic membranes in contrast to bare CA membrane undeniably assists in enhanced water permeability. (2) The introduction of GO/TiO_2_ nano-photocatalysts fabricates the membrane with more excellent hydrophilicity, accelerating the transfer of water molecules through the membrane surface [65]. (3) PVP is a hydrophilic biocompatible polymer. This increasing trend of water permeability is following the fashion of contact angle (Figure 9). Therefore, a more incredible hydrophilic nature, higher porosity, and more prominent finger-like channels of NC(3)-CA membrane contributed to its superior permeation performance over NC(2)-CA and NC(1)-CA membranes.

The rejection behavior of bare and hybrid membranes are also illustrated in Figure 10. By implying inorganic nanomaterials, viz. TiO_2_, GO, and its nanocomposite (GO/TiO_2_), the rejection of MO was thoroughly increased. The lowest value of rejection, approx 61.1%, was recorded for bare CA membrane, and the highest value of almost 96.6% was recorded for NC (3)-CA. This behavior might be credited to the small pore size combined with enhanced hydrophilic features [40]. The dye molecule present on the membrane surface was degraded by GO/TiO_2_ nano-composite through photocatalysis. This photodegradation of MO during the filtration phenomenon serves as the cherry on the top. Consequently, the rejection of pollutants is increased, membrane fouling restrained, and the flux of all hybrid membrane increased too. 

### 3.3. Photocatalytic Response of Nano-Photocatalytic Membranes

**Figure 11:**
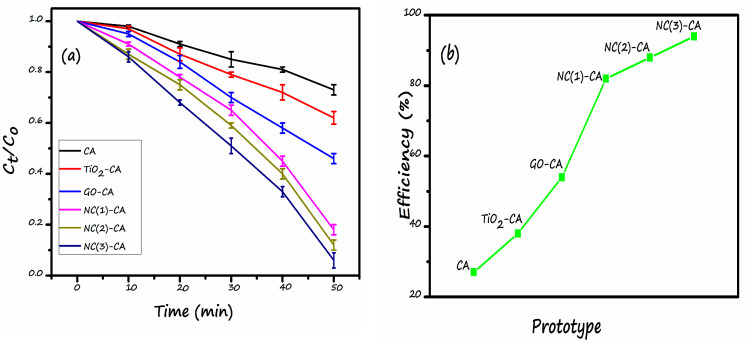
Photocatalytic assessment (**a**) Photodegradation of MO under visible light (**b**) Plot comparing the efficiency of various membrane.

The photocatalytic behavior of various membranes was evaluated under a visible light source (Xe-lamp). Firstly, the solution was kept in darkness for almost 45 min to establish adsorption-desorption equilibrium. Afterward, the light was turned on. Pristine CA-membrane almost showed no photocatalytic activity. It implies that the bare CA-membrane is incapable of illustrating appropriate photocatalytic activity. On the contrary, supplementing the single photocatalyst (TiO_2_ and GO) or nanocomposite photocatalyst (GO/TiO_2_) to the membrane matrix resulted in the greater photocatalytic response under visible light (Figure 11a). The photodegradation activity of TiO_2_-CA and GO-CA membrane was also investigated under similar conditions. Figure 11b demonstrates the photocatalytic efficacy order: NC(3)-CA (98.1%) > NC(2)-CA (91.6%) > NC(1)-CA (85.4%) > GO-CA (57%) > TiO_2_-CA (37%) >Pristine CA (25%). The photocatalytic activity of GO-CA (81%) and TiO_2_-CA (73%) is almost similar. This is because both semiconductors have almost similar band gaps and issues of charge carrier recombination, limiting the applicability of any photocatalysts correlated with both of them. Higher efficiency of GO-CA than TiO_2_-CA could be related to the visible light active band gap of GO compared to TiO_2_, a UV active photocatalyst, thus poor efficiency. Results illustrate that NC(3)-CA posessed the highest photocatalytic response. The highest response of nanocomposite photocatalytic membrane is owing to the two reasons: (1) Synergistic effect of TiO_2_ and GO. The integration of GO and TiO_2_ shift the bandgap of TiO_2_ somewhat into the visible region, as confirmed by the DRS spectra (Figure 6a). Thus, it utilizes a wide range of the spectrum. (2) Poor charge carrier recombination: GO forms heterostructure with TiO_2_. This configuration lowers the recombination process of charge carrier and leads to the superior photocatalytic response. The detailed mechanism is discussed in a later section. These findings are well-consistent with already published reports. For instance, Gao et al. reported the superior photocatalytic response of GO/TiO_2_-PVDF membrane is due to the synergistic effect of GO and TiO_2_ [39,66]. The varied amount of GO has a significant effect on the photocatalytic efficiency of the membrane. NC(3)-CA showed the maximum response because of the lowest band gap value of GO/TiO_2_ NC (2.99 eV) incorporated in the respective membrane, as shown in Table 2. This results in a greater adsorption of MO molecule on GO surface, and more active visible light absorption results in a greater photocatalytic response [67].

**Figure 12:**
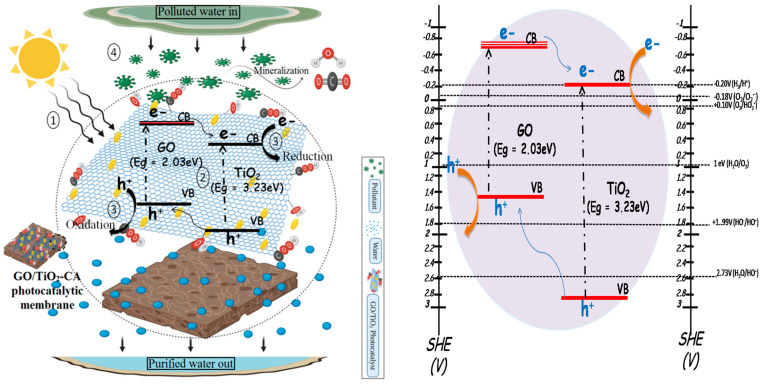
Possible mechanism of GO/TiO_2_-CA membrane for wastewater treatment.

Possible mechanism:

It is highly fascinating to look into the degradation mechanism of methyl orange (MO) by photocatalysis on the GO/TiO_2_-CA membrane surface (Figure 12). Literature studies reveal that GO can act both as an electron acceptor and a photosensitizer. Under UV light excitation, the charge carriers are produced by TiO_2_. Electrons from the conduction band of TiO_2_ transfer swiftly to the GO sheets. This phenomenon is supported by the more positive work function of graphene (4.42 eV) in contrast to the TiO_2_ CB (4.20 eV). The electrons scavenged by the graphene decrease the charge carrier recombination and elevate the photocatalytic response [68,69]. These electrons thereby reduce adsorbed oxygen present on the surface or dissolved in water and generate reactive oxygen species (OH and $O_{2}^{-}$), which further degrade the pollutant species. GO function as photosensitizer under visible light. The conduction band edge value of GO is −0.75 V, while the valence band edge lies at 1.5 V (vs. NHE) [70]. Similarly, the CB edge of TiO_2_ is lying at –0.2 V, and VB lies at 3 V. Consequently, the electrons migrate from the conduction band of GO to the CB of TiO_2_. The electron in the conduction band of TiO_2_ act on the MO molecule, adsorbed on the surface of membrane. In contrast, the holes from the valence band of TiO_2_ move towards the valence band of GO. Herein, the oxidation reaction of pollutants takes place. This antagonistic movement of electrons and holes lowers the recombination process Figure 12b. Briefly explaining the degradation mechanism explaining, Figure 12a shows (1) Adsorption of MO dye on the membrane surface followed by switch on the visible light: dye molecule adsorbs over the membrane owing to the presence of NH group in MO. It forms a hydrogen bond with the hydroxyl groups of the membrane, thus establishing chemical bonding [71]. (2) The electron and holes are generated. (3) Generation of ROS. (4) Redox reaction takes place, thereby mineralizing MO to hydrazine derivative. Different redox reactions led to the production of CO_2_ and H_2_O. We believe, in this study, that the model pollutant is degraded through mechanism 2. However, this needs to be investigated further through XPS and ESR studies. Wang et al. fabricated Cu_2_O, TiO_2_/rGO heterojunction PM. Under UV-Vis light, the membrane exhibit outstanding performance due to the synergistic effect of Cu_2_O, TiO_2_ and rGO in heterojunction [72]. Recently, Yian Chen et al. reported the synthesis and photocatalytic performance of cellulose/GO/TiO_2_ hydrogel. The higher photocatalytic activity of composite hydrogel was associated with the strong coupling between TiO_2_ and GO, which lowers the recombination process. Hence, it has enhanced the photocatalytic and absorption performance of hydrogel [73].

### 3.4. Anti-Fouling and Self-Cleaning Assessment of Various Membranes

**Figure 13:**
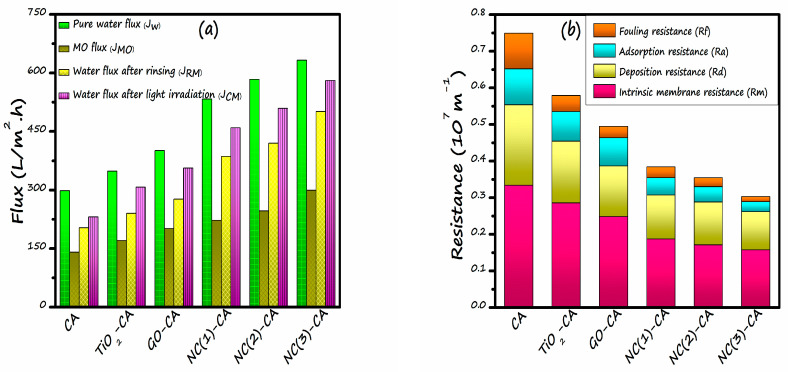
(**a**) Flux calculated at the various processes (pure water flux, MO flux, water flux after rinsing, and water flux after light irradiation), (**b**) Evaluation of the filtration resistances for different membranes.

The anti-fouling character of all the pristine and hybrid membranes was investigated by a four-step filtration procedure as described in Section 2.6.2. Figure 13 illustrates the MO soln. equilibrium flux measurement of all the membranes before and after water rinsing and light irradiation. In contrast to pure water flux (*J_w_*), the MO solution flux (*J_MO_*) decreased significantly. It happened due to membrane fouling occurred through the adsorption as well as deposition of dye molecules on the surface of membrane. When the membrane was rinsed with water, the flux values were recovered to different degrees due to the eradication of foulant, loosely bonded to the membrane through mere shear force. Following water cleaning, the membrane was exposed to the light source. In this way, the foulants firmly attached to membranes were removed, and a further increment of flux was observed for all hybrid membranes. However, the maximum increase in flux after light irradiation (*J_CM_*) was observed for NC(3)-CA membrane owing to the more fantastic photocatalytic performance and photo-induced hydrophilicity of GO(2 wt%)/TiO_2_ nanocomposite. These two properties prove beneficial for the degradation of strongly bound foulants and bestow the membrane with a favorable anti-fouling property and self-cleaning ability. Despite the increment of fluxes with the light irradiation and washing, the fluxes still failed to recover completely. This finding accords with the work of M. Tavakol Moghadam [74].

To further investigate the self-cleaning ability of the membrane, various filtration resistances are calculated as illustrated in Figure 13b. The total resistance (*R_t_*) of all nanocomposite employed membranes, viz. NC(1)-CA, NC(2)-CA, and NC(3)-CA, is smaller than the bare CA, GO-CA, or TiO_2_-CA membrane, suggesting greater water flux as well as reduced fouling after the filtration of MO solution.

- *R_m_* is designated as intrinsic membrane resistance. Its value corresponds to the porosity of the membrane. However, *R_m_’s* value decreases with the introduction of nano-photocatalysts (GO, TiO_2_, and GO/TiO_2_). This trend is in accordance with the results of membrane porosity [43].
- Deposition resistance (*R_d_*) is strongly related to the hydrophilicity of the membrane. R_d_ happens because of cake layer formation on the surface of membrane. *R_d_* decreases with the decrement in the contact angle. In other words, the value of deposition resistance decreases with the increase of hydrophilicity of the membrane. It employs that the *R_d_* value of NC(3)-CA membrane is lowest.
- Adsorption resistance (*R_a_*) occurs due to the strongly bound pollutant molecules on the membrane surface. These contaminants are merely removed by water rinsing, can only be removed by the photodegradation phenomenon. This resistance is highly dependent on the self-cleaning ability of the membrane. Under the light, the *R_a_* value decreases sharply due to the self-cleaning mechanism and improvement in hydrophilicity. NC(3)-CA membrane showed the minimum *R_a_* value, suggesting superior self-cleaning ability.
- The fouling resistance (*R_f_*) of all nanocomposite-employed membranes is lesser than the bare membrane on account of the excellent hydrophilicity and photodegradation of foulant (MO dye) at the periphery and inside the pores. However, the NC(3)-CA membrane showed the most negligible *R_f_* value.

**Figure 14:**
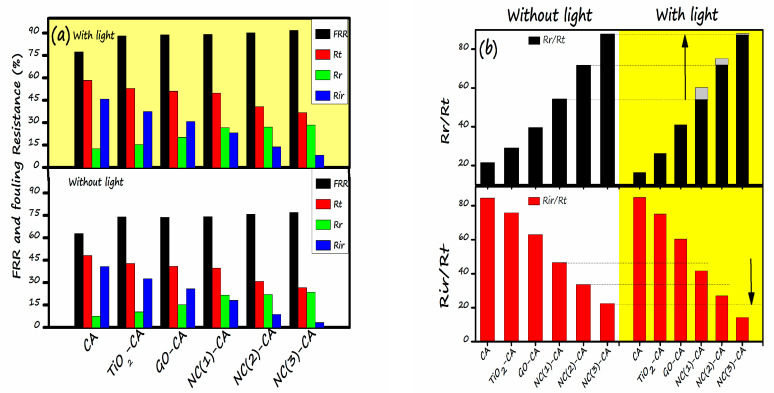
(**a**) Influence of light on FRR and fouling resistances of various membranes (**b**) Influence of light on the ratio of reversible fouling and irreversible fouling to total fouling of various membrane.

To further observe the fouling of membrane, two essential parameters viz. Flux recovery ratio (*FRR*), Fouling ratio (*Fr*) was calculated by employing Equations (12) and (13) respectively are presented in Figure 14. More often than not, a more excellent FRR value represents a superior anti-fouling property of membrane. The *FRR* value for all the membranes supplemented with NC(1)-CA, NC(2)-CA, and NC(3)-CA is higher than the bare CA, GO-CA, or TiO_2_-CA membranes, representing the enhanced anti-fouling performance of nanocomposite employed membranes. Without light, the FRR value of the bare CA membrane is 62.98%, lowest amongst all other membranes, indicating poor anti-fouling capability. In contrast, the highest *FRR* (76.99%) was noticed for NC(3)-CA. This might be ascribed to the improvement in hydrophilicity of the membrane. When light is turned on, an amelioration in the anti-fouling behavior was noticed for all the membranes. The increment in the *FRR* value is due to the photocatalytic degradation of MO under the light. In the best-case scenario, the *FRR* value for NC(3)-CA membrane was increased from 76.99% to 91.78%. The increment in the *FRR* value with light justifies the self-cleaning ability of the membrane, implying good agreement with the findings of reference [45,74]. Moreover, the fouling ratio (*Rt*) (reversible fouling (*Rr*) + irreversible fouling (*Rir*)) of all the membranes followed the sequence CA > TiO_2_-CA > GO-CA > NC(1)-CA > NC(2)-CA > NC(3)-CA. The result demonstrates that the fouling ratio of nanocomposite membrane is the lowest. Moreover, results signify that the improvement in hydrophilicity, the introduction of the nanocomposite, and the light source are critical factors determining the membranes’ enhanced anti-fouling ability. In depth analysis reveals that the occurrence of membrane fouling might be due to the contribution reversible resistance and irreversible resistance. The reversible resistance takes place owing to the dye molecule adsorption on membrane surface. Figure 14b demonstrates the reversible fouling to total fouling percentage (*Rr/Rt*) and irreversible to total fouling percentage (*Rir/Rt*) of all the photocatalytic membranes. The reversible fouling to total fouling percentage (*Rr/Rt*) was increased for hybrid membranes in the presence of light. On the contrary, irreversible to total fouling percentage (*Rir/Rt*) of the hybrid membranes decreased in the presence of light compared to the membranes without light. The overall membrane fouling for the hybrid membranes is dominated by irreversible to total fouling percentage (*Rir/Rt*), and could be mitigated with light due to the photocatalytic degradation of dye molecules. Generally, hybrid membranes showed enhanced antifouling photocatalytic performance. Therefore, the NC(3)-CA membrane outperforms the antifouling field.

Table 3 presents some reported high-performance photocatalytic membranes. It illustrates that the current research with phenomenal photocatalytic performance and excellent flux recovery ratio may widen the domain of graphene-based visible-light-driven photocatalytic membranes. Moreover, nanocomposite membranes possess great potential for wastewater treatment due to the photodegradation of contaminants on the membrane surface. In this work, for the first time, GO(x wt%)/TiO_2_-CA were fabricated and evaluated.

## 4. Conclusions

In summary, a novel NC(x)-CA nano-photocatalytic membrane was synthesized by employing a simple, facile, and cost-effective phase inversion technique by integrating GO (0.5, 1, 2 wt%)/TiO_2_ nano-photocatalysts into the CA membrane matrix. Various characterization techniques, viz. XRD, FT-IR, EDX, DRS, and SEM, contact angle values confirmed the successful incorporation of photocatalysts into the CA membrane matrix. The experimental studies reveal that all the nanocomposite photocatalytic membranes exhibit superior performance compared to pristine CA and single photocatalyst membranes (GO-CA and TiO_2_-CA) owing to the synergistic effect of GO and TiO_2_. However, the NC(3)-CA membrane with 2 wt% GO content exhibits an ideal morphology (SEM image: Figure 8f), high porosity (as confirmed by BET analysis), improved hydrophilicity (lowest contact angle value: 49.1°: Figure 9), highest pure water permeability (613(L/m^2^h: Figure 10), greater photodegradation efficiency (98.1%: Figure 11), superior flux recover ratio (91.78%: Figure 14), as well as lowest total and fractional resistances (Figure 14). This might be attributed to the robust and synergistic photocatalytic activity of NC-3 (as explained through mechanistic approach (Figure 12)) which endows the NC(3)-CA membrane with superior self-cleaning ability and anti-fouling performance over all other composite and bare membranes. Thus, the NC(3)-CA membrane possesses excellent potential to be considered the next generation membrane in the field of wastewater treatment.

## Figures and Tables

**Table 1:** The composition of membranes.

Membrane Sample	CA (wt%)	PVP (wt%)	NMP (wt%)	Additives	
				Nanomaterials	Amounts (wt%)
Pure (CA)	16	2	82	-	0
TiO_2_-CA	16	2	81	TiO_2_	1
GO-CA	16	2	81	GO	1
GO/TiO_2_-CA	16	2	81.5	GO/TiO_2_-x	0.5
GO/TiO_2_-CA	16	2	81	GO/TiO_2_-x	1
GO/TiO_2_-CA	16	2	80	GO/TiO_2_-x	2.0

**Table 2:** Table represents the EDX-% composition, avg. The crystallite size (nm), avg. particle size (nm), Bandgap (eV), and surface area (m^2^/g) of all the prepared samples.

S#	Sample Code	EDX-Percentage Composition			XRD-Avg. the Crystallite Size (nm)	SEM-Avg. Particle Size (nm)	DRS-Band Gap (eV)	BET-Surface Area (m^2^/g)
		Atomic % of Ti	Atomic % of O	Atomic % of C				
1	**TiO_2_**	13.56	86.44	----	20.9	36.33	3.23	11
2	**GO**	----	----	100	----	----	2.08	----
3	**NC-1** **(0.5:1 GO-T)**	20.42	36.90	42.55	13.7	33.93	3.19	57.49
4	**NC-2** **(1:1 GO-T)**	5.97	51.85	42.11	13.6	32.77	3.14	62.08
5	**NC-3** **(2:1 GO-T)**	2.48	25.79	71.63	13.3	32.55	2.99	63.23

**Table 3:** Comprehensive comparison of various published nanomaterials incorporated membranes with this study.

S.#	Membranes	Synthesis Method	Pollutant/Foulant	Photocatalytic Activity (%)	Permeation Flux Recovery Ratio (%)	Year	(Ref.)
1	Silica/Titania-Al_2_O_3_ (Pioneer work)	Sol-gel method	Direct black 168	85%	-	2006	[20]
2	PVDF-NZPs	In-situ reduction method.	BSA, yeast	-	83% (both)	2020	[75]
3	NGO/TiO_2_-PSF	NIPS	MB	Sunlight-77.5	Sunlight-90.1%	2018	[57]
4	GO/ZnO-PVDF	Immersion-precipitation phase transformation	MB	86.84%	-	2019	[76]
5	ZnIn_2_S_4_-PVDF	Phase inversion and deposition	Fluvastatin (photocatalytic application) RhB (Antifouling application)	97.19	76.58%	2020	[77]
6	MCU-C_3_N_4_/PVDF	Vaccum filtration	RhB, TC	84.24%, 71%	91%	2019	[78]
8	GO/TiO_2_-PVDF	Phase inversion technique	BSA	80%	82.1%	2016	[44]
9	g-C_3_N_4_ NS/RGO-CA	Vacuum filtration method	RhB	60%	-	2016	[79]
10	P-CND/TiO_2_ -PVDF	NIPS	HA	-	80%	2020	[80]
11	Au0.1Ag0.9/TiO_2_/CA	Phase-inversion method	Tetracycline (TC)	90%	-	2019	[81]
12	GO(0.5 wt%)/TiO_2_-CA	NIPS	MO	85.4%	89.18%	2021	This study
	GO(1 wt%)/TiO_2_-CA			91.6%	90.28%		
	GO(2 wt%)/TiO_2_-CA			98.1%	91.78%		
